# Anticancer Activity of 2α, 3α, 19β, 23β-Tetrahydroxyurs-12-en-28-oic Acid (THA), a Novel Triterpenoid Isolated from *Sinojackia sarcocarpa*

**DOI:** 10.1371/journal.pone.0021130

**Published:** 2011-06-10

**Authors:** Ouchen Wang, Sujun Liu, Jiawei Zou, Liting Lu, Lin Chen, Sunquan Qiu, He Li, Xincheng Lu

**Affiliations:** 1 Institute of Genomic Medicine, Wenzhou Medical College, Wenzhou, China; 2 First Affiliated Hospital of Wenzhou Medical College, Wenzhou, China; 3 Leshan Normal University, Leshan, China; Bauer Research Foundation, United States of America

## Abstract

**Background:**

Natural products represent an important source for agents of cancer prevention and cancer treatment. More than 60% of conventional anticancer drugs are derived from natural sources, particularly from plant-derived materials. In this study, 2α, 3α, 19β, 23β-tetrahydroxyurs-12-en-28-oic acid (THA), a novel triterpenoid from the leaves of *Sinojackia sarcocarpa*, was isolated, and its anticancer activity was investigated both *in vitro* and *in vivo*.

**Principal Findings:**

THA possessed potent tumor selected toxicity *in vitro*. It exhibited significantly higher cytotoxicity to the cancer cell lines A2780 and HepG2 than to IOSE144 and QSG7701, two noncancerous cell lines derived from ovary epithelium and liver, respectively. Moreover, THA showed a dose-dependent inhibitory effect on A2780 ovary tumor growth *in vivo* in nude mice. THA induced a dose-dependent apoptosis and G2/M cell cycle arrest in A2780 and HepG2 cells. The THA-induced cell cycle arrest was accompanied by a downregulation of Cdc2. The apoptosis induced by THA was evident by induction of DNA fragmentation, release of cytoplasmic Cytochrome c from mitochondria, activation of caspases, downregulation of Bcl-2 and upregulation of Bax.

**Conclusion:**

The primary data indicated that THA exhibit a high toxicity toward two cancer cells than their respective non-cancerous counterparts and has a significant anticancer activity both *in vitro* and *in vivo*. Thus, THA and/or its derivatives may have great potential in the prevention and treatment of human ovary tumors and other malignancies.

## Introduction

Natural products represent a rich reservoir for anticancer compounds [1], [2]. Over sixty percent of currently used anticancer drugs are derived from natural sources such as plants, marine organisms and microorganisms [3], [4]. In particular, higher plants have provided many effective, clinically useful anticancer drugs. For examples, the vinca bisindole alkaloids, the epipodophyllotoxin analogs, the taxanes, and the camptothecins, are all effective plant-derived anticancer drugs [5],[6].

In the past decades, there is growing interest in another group of natural compound: the triterpenoids. Triterpenoids are structurally diverse organic compounds of more than 20,000 naturally occurring variants that share a basic triterpenoid moiety [7], [8]. These compounds are ubiquitously distributed throughout the plant kingdom and have many biological activities. For examples, several triterpenoids, including saponin, oleanolic acid, betulinic acid, celastrol, pristimerin, lupeol, and avicins have been reported to possess anti-inflammatory, hepato-protective, or antitumor properties [9], [10]. In particular, ursolic acid has been shown to constitute a main active component in a number of oriental and traditional medicine herbs with a variety of biological activities including induction of differentiation, anti-proliferation, and antitumor activities [11]–[13]. Mechanistically, triterpenoids have been implicated in a wide range of biological pathways or processes including nuclear factor-kappaB signalling, apoptosis, transcription, angiogenesis, and tumor metastasis [14]–[16]. Thus, these compounds are potential candidates for anticancer agents.

Recently, a number of compounds with anti-proliferative and/or antitumor activities have been isolated from plants of the *Styracaceae* family, suggesting that this family of plants may be a new source for compounds with anticancer activities [17]–[23]. *Sinojackia sarcocarpa* is a member of the *Styracaceae* family [24]. It consists of primarily deciduous arbor trees that are distributed in the subtropical zone of the Eastern and Southern China, and was once at the brink of extinction. Intriguing, this endangered status has prompted a great effort in the development and optimization of propagation and cultivation technologies for these plants. As a result, it represents an attractive source for raw materials of its family. Here, we report the identification of 2α, 3α, 19β, 23β-tetrahydroxyurs-12-en-28-oic acid (THA), a new triterpenoid molecule from *Sinojackia sarcocarpa*. More importantly, we have shown that THA exhibited a significantly higher cytotoxic effect towards an ovarian cancer cell line and a liver cancer cell line than their respective noncancerous counterparts. The compound also elicited a G2 arrest and a significant apoptotic response. It was well tolerated and exhibited significant antitumor activity in nude mice.

## Methods

### Reagents

Tissue culture plasticware was purchased from BD Biosciences (USA). Column chromatography (silica gel 60), HPLC grade acetonitrile and dimethylsulphoxide (DMSO) were from Merck (Germany). Propidium iodide (PI) was product of Sigma (USA). The broad-spectrum caspase inhibitor z-VAD-fmk, anti-human p21 (C-19), Bcl-2 (100), Bax (N-20), procaspase-3 (N-19), procaspase-8 (8CSP03), procaspase-9 (4i31), Cyclin D1 (DCS-6), Fas (B-10), Cdc2 (B-5), and PARP (H-250), together with all secondary antibodies (anti-mouse, anti-goat and anti-rabbit immunoglobulin G) were purchased from Santa Cruz Biotechnology, Inc. (USA). The anti-human Cytochrome c (4272) and beta-actin (13E5) antibodies were from Cell Signaling Technology, Inc. (USA). Anti-c-Myc (K422) was from Bioworld Technology, Inc. (USA). Cell culture media, fetal bovine serum (FBS), phosphate-buffered saline (PBS), sodium pyruvate, non-essential amino acids, penicillin/streptomycin and other cell culture supplies were obtained from Invitrogen (USA).

### Cell culture

The Immortalized non-tumorigenic human ovarian surface epithelial cells IOSE144 and human cancer cell lines HCT116, HepG2, A2780, A549, PC3, MCF-7 and Eca109 were originally obtained from the American Type Culture Collection (ATCC), QSG7701 and other cell lines used in this study were from the Cell Resources Centre of Shanghai Institute for Biological Sciences, Chinese Academy of Sciences (China). Cells were grown in RPMI-1640 or DMEM, with 10% FBS, 1% penicillin/streptomycin solution (100 IU/ml penicillin, 100 mg/ml streptomycin) and 2 mM L-glutamine in a 5% CO_2_ atmosphere at 37°C.

### THA preparation

The plant leaves of *Sinojackia sarcocarpa* were collected from the forest in Sichuan, China. The isolation process of THA was demonstrated in the Supporting information (Fig. S1). Briefly, the dried leaves of *Sinojackia sarcocarpa* (10 kg) were extracted with 20 liter of 95% ethanol for 3 hour at room temperature and then subjected to sonication. The material was concentrated with vacuum to obtain a viscous extract of approximately 150 g. This extract was suspended in water and extracted first with petroleum ether and then EtOAc. This yielded approximately 20 grams of EtOAc extract. The EtOAc extract (20 g) was further fractionated by a silica gel column chromatography into 20 fractions. Finally, fraction 6 (4.0 g) was subjected to HSCCC separation using a standard hexane–ethyl acetate–methanol/water (10∶5∶3∶1, v/v) two-phase solvent procedure, yielding 460 mg final dried powder. The structure of the major compound in this powder was determined based on data from UV, IR, MS, ^1^H NMR, and ^13^C NMR spectra analyses.

### Cell viability and clonogenic survival assays

The cell viability was determined by MTT assay. Briefly, 2∼5×10^4^ cells per well were plated in triplicate in 96-well plates. After 24 hr incubation at 37°C in 5% CO2, the cells were treated with the THA at following concentrations: 2.5, 5, 10, 20, 40, 80 and 160 µg/ml, a DMSO treated group was kept as control. After incubation for 72 hr, 10 µl of the 3-(4,5-dimethylthiazol-2-yl)-2,5-diphenyltetrazolium bromide (MTT, Sigma, USA) stock solution (5 mg/ml) was added into each well. The plates were incubated in 37°C, 5% CO2 for another 4 hr. The medium was carefully removed from each well and 100 µl of DMSO was added. The plates were gently agitated until the color reaction was uniform and absorbance of the converted dye was measured at a wavelength of 550 nm with Microplate Reader (BioRad Model 550). Microsoft-Excel 2000 was used for data analysis. The percentage of viability was calculated as: % Viability = [OD of treated cells/OD of control cells]×100. The IC_20_, IC_50_, IC_80_ were defined as the THA concentrations that reduced the absorbance of the treated wells by 20%, 50%, 80% as compared with the control cells. To verify that THA does not react with the MTT assay reagents, we also performed the MTT assay in the absence of cells. The clonogenic survival assay was modified from the previous report [25]. Briefly, cells were plated at a low density (500 viable cells per well) in triplicate in 6-well culture plates without (control, with DMSO) or in the presence of the THA. Seven days later, the cells were fixed with 2% formaldehyde in PBS and stained with hematoxylin, and colonies of more than 20 cells were scored.

### Detection of apoptosis

The cells were plated in 6-well plate at a seeding density of 5×10^5^ cells/well and treated with different concentrations of THA (0, 5, 10, 20, 40 µg/ml). After 48 hr of treatment, the cells were harvested and washed with PBS twice. The floating and trypsinized adherent cells were collected and stained with Annexin V-FITC (Invitrogen, USA) following the manufacturer's instructions. Samples were analyzed with a FACSAria™ flow cytometer (Becton Dickinson, USA) after incubation in the dark at room temperature for 5 min. Cells positive for early apoptosis (Annexin V-FITC stained only, see Q4 in figure) and for late apoptosis (Annexin V-FITC and PI stained, see Q2 in figure) were combined.

### DNA fragmentation analysis

The appearance of DNA fragmentation was determined by agarose gel electrophoresis [26]. The cells were treated as above and washed with cold PBS. The cell pellets were lysed with 2% SDS containing 10 µg/ml RNase A and incubated at 37°C for 2 hr. 5 M NaCl was added to a final concentration of 1 M, and cells were scraped and stored at 4°C for 24 hr. The lysed cells were centrifuged for 30 min at 12,000 rpm. DNA unassociated with intact chromatin residing in the supernatant was extracted by phenol-chloroform and precipitated with ethanol. DNA (10 µg) was resolved by agarose gel electrophoresis in 1×Tris-acetate-EDTA for 1 hr on 1.5% agarose gel impregnated with ethidium bromide.

### Cell cycle measurements

5×10^5^ A2780 and HepG2 cells were seeded in 6-well plates and allowed to adhere overnight and then the medium was removed and replaced with fresh medium containing 0, 10, 20 and 40 µg/ml concentrations of the THA and 1% FBS. After 48 hr of incubation, the cells were harvested by centrifugation at 1000 rpm for 5 min. The cell pellets were washed twice with PBS followed by fixation with ice-cold 70% ethanol and storage at −20°C overnight. After centrifugation, the pellets were washed with cold PBS, suspended in 500 µl PBS with 50 µg/ml PI (final concentration), 0.1 mg/ml RNase A, 0.05% Triton X-100, and incubated at 37°C for 40 min in the dark. The cell cycle distribution was determined on the Becton Dickinson FACSCaliburr. The experiment was repeated thrice under the same conditions.

### Western blot analysis

Cell extracts were prepared from control as well as cells treated with different concentrations of THA (0, 5, 10, 20, 40 µg/ml). Briefly, the cells were washed twice with ice-cold PBS and scraped into 100 µl lysis buffer containing 50 mM Tris (pH 7.4), 5 mM EDTA, 0.5% NP-40, 1 mM PMSF, 150 mM NaCl and incubated on ice for 20 min with intermittent mixing. The extract was centrifuged for 20 min at 4°C at 12000 rpm. Cytosolic proteins were prepared using a nuclear/cytosol fractionation kit (Pierce, USA). Equal amount of protein was loaded on a 10–12% SDS-PAGE gel and transferred electrophoretically to Amersham Hybond-P PVDF membrane in sodium phosphate buffer. The membrane was blocked in 5% milk in PBST and incubated at room temperature for 2–4 hr with first antibody. After three washings with 0.1% Tween-20 in TBS, the membrane was incubated for 2 hr at room temperature with HRP-conjugated secondary antibody. Protein bands were visualized by the ECL system (Amersham Biosciences, USA).

### Xenograft growth assay

Pathogen-free female athymic nude mice (nu/nu, 4–6 weeks) were purchased from Shanghai Experimental Animal Centre (Shanghai, China). All animals were fed with commercial diet and all animal studies were approved by the Wenzhou Medical College, Institutional Animal Care and Use Committee (approval ID WAC-2009003). The xenograft model was established as reported [27]. Briefly, A2780 cells were harvested and resuspended in serum-free RPMI 1640 medium containing 20% (v/v) Matrigel (BD Biosciences, USA). Aliquots of cells (5×10^6^ cells/0.2 ml) were injected subcutaneously into the inguinal region of nude mice. Mice bearing palpable tumors (∼50 mg) were randomly divided into treatment and control groups (n = 5 mice/group). THA was diluted in saline and was administered via i.p. injection at doses of 25 and 50 mg/kg (inject once every other day for 3 weeks). The control group received saline only. The tumor growth and body weight of the mice were monitored every other day. Tumor sizes were determined using calliper measurement and tumor volumes (mm^3^) were calculated using the standard formula: length×width×height×0.5326. Tumors were harvested 3 weeks after treatment and individually weighed.

### Statistical analysis

All data represent three independent experiments and are expressed as the mean ± SD unless otherwise indicated. Statistical comparisons were made by two-tailed Student's t-test. Significance was considered as P<0.05.

## Results

### Isolation of cytotoxic compounds from *Sinojackia sarcocarpa*

**Figure 1:**
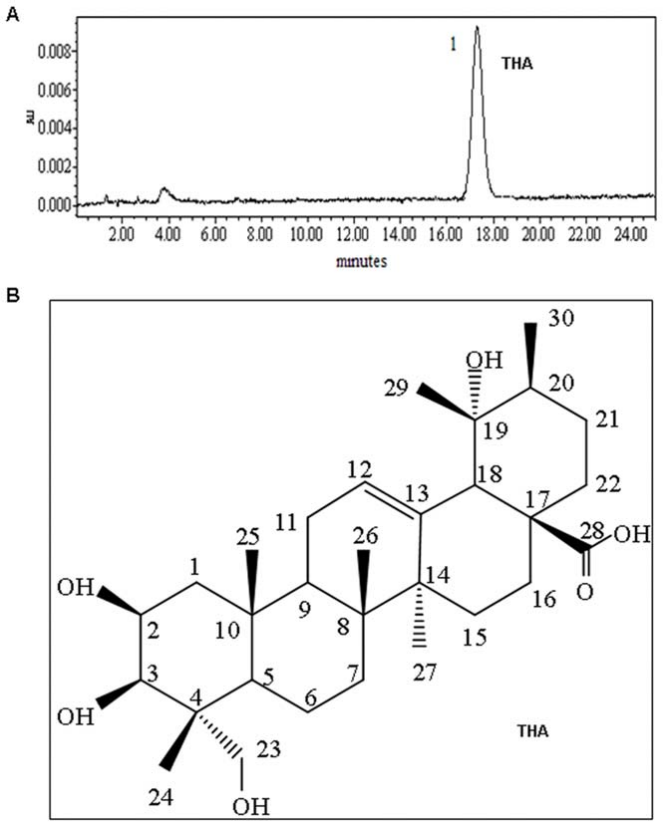
Chemical structure and purity of THA. (A) The purity of THA was determined by HPLC. (B) The chemical structure of THA was identified as 2α, 3α, 19β, 23β-tetrahydroxyurs-12-en-28-oic acid.

A two-step extraction procedure was first used to obtain an extract enriched in alkaloids and flavanoids. This extract was then fractionated by column chromatography into 20 fractions. The purification process is illustrated in Figure S1. Individual fractions from the column chromatography were then used and tested for their cytotoxic effect on the colon cancer cell line HCT116. The results from preliminary experiments showed that Fraction 6 exhibited the most profound cytotoxic effect on HCT116 cells. Thus, Fraction 6 was further purified by HSCCC, resulting in a total of 460 mg dried powder (from a 10 kg starting raw material). HPLC analysis revealed that this powder contained primarily a single compound with 97.97% purity (Fig. 1A). The identity of the major constituent of the powder was then determined by a series of analyses including IR absorptions, ^1^H NMR spectrum, ^13^C NMR spectra analysis. The data from these analyses in aggregate indicated that the compound was 2*α*, 3*α*, 19*β*, 23*β*-tetrahydroxyurs-12-en-28-oic acid (THA) (Fig. 1B) and that it has a molecular formula of C_30_H_48_O_6_ and molecular weight of 504.35. To our knowledge, this is a novel triterpenoid that has not been reported in the literature previously.

### THA inhibits cancer cell growth *in vitro*

**Figure 2:**
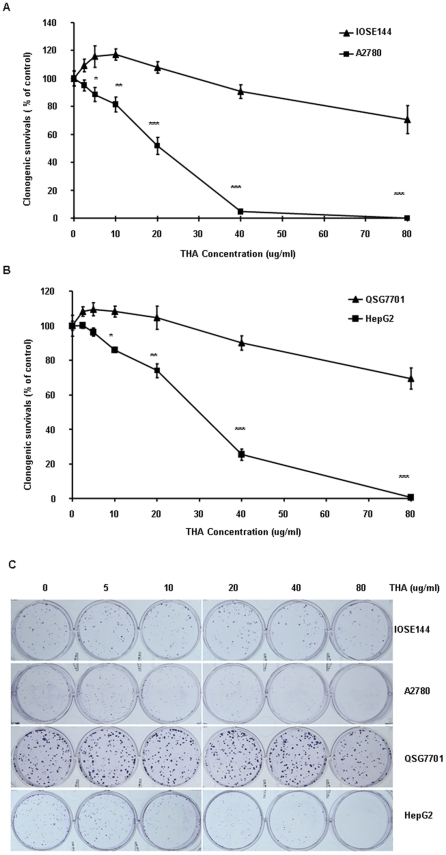
THA is much more potent in inhibiting the clonogenic survival ability of cancerous cells versus their noncancerous counterparts. (A) Ovary cancer cell A2780 and ovary epithelial cell IOSE144. (B) Hepatocelluar carcinoma cell HepG2 and normal liver cell QSG7701. (C) The photos of clonogenic growth assay. Note the trend of the pronounced increase in growth suppression in the two cancer cell lines as the concentration of THA increases (from left to right). Cells were exposed to various concentrations of THA for seven days followed by clonogenic assay. All assays were done in triplicate (*, P<0.05; **, P<0.01; ***, P<0.001 versus noncancerous counterparts).

A preliminary study indicated that THA did not react with the MTT assay reagents in the absence of cells (Fig. S2).Therefore, MTT assay was used to assess the anti-proliferation activity of this compound. Fifteen cell lines representing ten types of human malignancies (liver, ovary, lung, gastric, esophageal, breast, prostate, gliomas, colon and thyroid) were cultured with THA at concentrations in the range of 2.5∼160 µg/ml for 72 hr, and a strong reduction in cell viability was observed in a dose-dependent manner. The concentrations that reduced growth by 20% (IC_20_), 50% (IC_50_), and 80% (IC_80_) are summarized in Table 1. An important attribute of an effective anticancer drug is cancer selectivity in its cytotoxic effect. Thus, having succeeded in purifying a cytotoxic triterpoid from *Sinojackia sarcocarpa*, we asked whether it exhibited any cancer selectivity in cell killing. First, we compared the cytotoxic effect of this agent on two different pairs of noncancerous-cancerous cell lines: 1) the noncancerous QSG7701 hepatocyte line and the HepG2 hepatocellular carcinoma line; 2) the noncancerous IOSE144 ovarian epithelial line and the A2780 ovarian cancer line, respectively. The results of the initial MTT assay experiments showed that in both cases, THA was significantly more toxic to the cancerous cell lines than to their noncancerous counterparts (Table 1). Clonogenic survival experiments also demonstrated that THA is much more potent in inhibiting the clonogenic survival ability of the cancer cells than their noncancerous counterparts (Fig. 2A, 2B, 2C). These results, therefore, suggest that THA exhibits a certain level of cancer selectivity in terms of its cytotoxic effect under our *in vitro* tissue culture condition.

**Table 1:** Growth inhibitory activity of the THA compound on different cancer cell lines.

Cell lines	Inhibitory concentration (µg/ml)		
	IC_20_	IC_50_	IC_80_
QSG7701	67.5	>100	>100
HepG2	14.2	29.7	52.0
IOSE144	60.0	>100	>100
A2780	11.2	24.3	34.8
A549	12.0	25.0	72.0
H1299	20.0	50.0	76.5
AGS	20.1	28.6	37.8
Eca109	6.3	26.0	36.0
CaEs17	15.2	27.0	38.7
MCF-7	37.5	55.0	72.0
MB-MDA-231	44.0	59.2	75.0
PC3	13.8	29.5	37.0
SHG-44	6.0	31.0	59.0
U251	20.3	33.7	63.6
HCT116	7.2	19.5	34.5
ARO	21.6	25.0	29.5
FRO	23.9	29.6	36.4

IC_20_, IC_50_, and IC_80_ were the concentrations of drug that inhibit growth by 20%, 50%, and 80%, respectively, relative to the control.

### THA possesses antitumor activity *in vivo*

**Figure 3:**
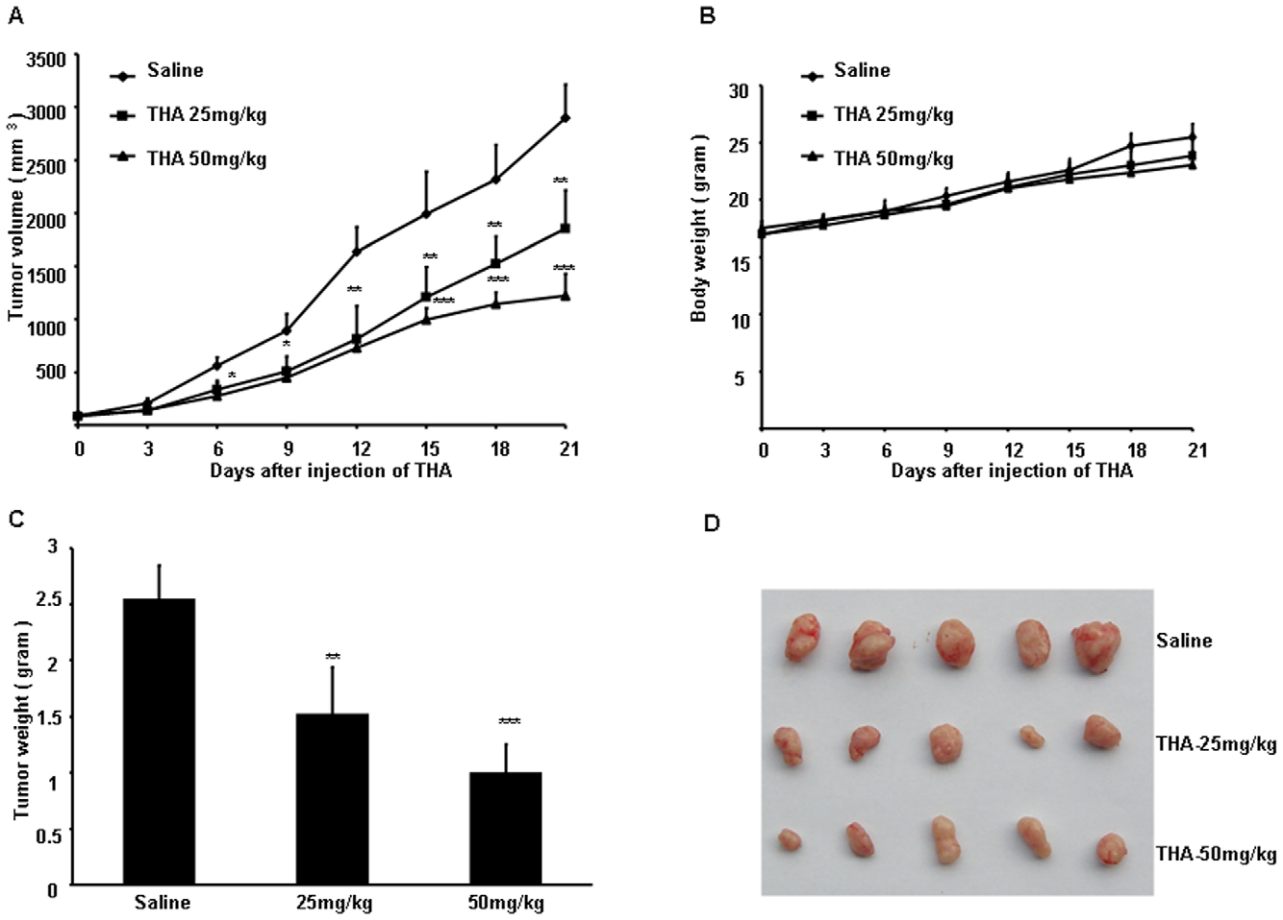
THA significantly inhibits ovary tumor growth in A2780 xenograft tumor model. (A) Time course of tumor growth at the indicated THA concentrations in mice bearing A2780 tumor. (B) The corresponding body weight changes during the THA treatments. (C) The tumor weights measured at autopsy after three weeks of THA treatment. (D) The photo of the autopsied tumors after three weeks of THA treatment (*, P<0.05; **, P<0.01; ***, P<0.001 versus saline control).

The selective killing property of THA suggests that it could have antitumor activity, i.e. suppressing the growth of tumors without inflicting a serious adverse effect on the host. To address this important issue, we assessed the potential antitumor activity of THA. A preliminary study indicated that a three-week course of daily i.p. injection of THA at concentrations as high as 50 mg/kg body weight did not cause any obvious adverse effects on either the daily food intake and body weight of mice (data not shown), suggesting that low doses of THA (50 mg/kg body weight or below) are well tolerated in mice. We then asked whether such a “non-toxic” doses of THA could have any antitumor activity *in vivo* using an A2780 xenograft tumor model. THA was administered via i.p. injection every other day for 3 weeks to mice in the treatment groups. Therapeutic effects were evaluated by examining tumor size and tumor weight. Consistent with the *in vitro* findings, THA showed a dose-dependent inhibitory effect on tumor growth (Fig. 3A). THA (at doses of 25 and 50 mg/kg) resulted in 39% (P<0.01) and 61% (P<0.001) tumor growth inhibition (compared to control mice treated with saline) in the A2780 xenograft tumor model (Fig. 3C, 3D). At this dose range, the agent did not cause a significant impact on the body weight or any other vital signs of the animals (Fig. 3B). Thus, within the “non-toxic” range, THA exhibited a significant antitumor activity to xenograft tumors derived from the A2780 ovarian cancer cell line.

### THA induces cell apoptosis

**Figure 4:**
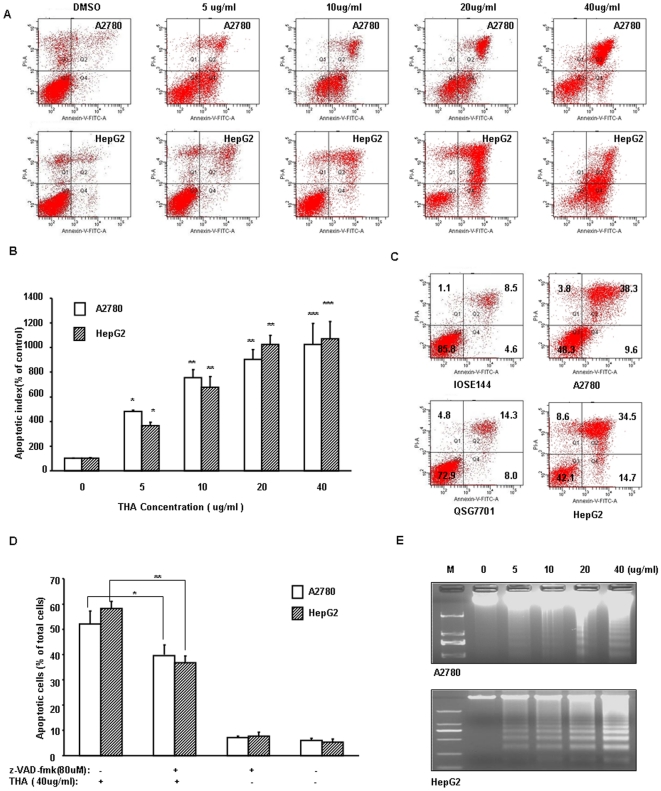
THA induces dose-dependent apoptosis in human ovarian and hepatocelluar carcinoma cells. (A) Cell apoptosis analyzed with Annexin V-FITC kit, A2780 and HepG2 cells were treated with DMSO or the indicated concentration of THA for 48 hr. (B) Apoptosis index of A2780 and HepG2 cells. Data are representative of the values from three independent experiments. Cells positive for early apoptosis and for late apoptosis were combined, and the percentage of cellular apoptosis in control cells was regarded as 100%. (C) Comparison of the cell apoptosis induced by THA in the cancerous A2780, HepG2 cell and noncancerous cells IOSE144 and QSG7701. Cells were treated with 30 µg/ml THA for 48 hr. (D) z-VAD-fmk partially prevented the THA induced apoptosis in A2780 and HepG2 cells. Cells were treated with 80 µM z-VAD-fmk for 1 hr before adding THA(40 µg/ml) for another 48 hr. (E) Dose-dependent effects of THA on DNA fragmentation in A2780 and HepG2 cells visualized by gel electrophoresis. (*, P<0.05; **, P<0.01; ***, P<0.001 versus the control, respectively).

Previously, it has been reported that some triterpenoids could induce apoptosis in cultured human cancer cells [28], [29]. Thus, we also examined whether THA could induce apoptosis in cancer cells. After treating the A2780 and HepG2 cells with different doses of THA, the percent of apoptotic cells was assessed by Annexin V-FITC and propidium iodide staining, followed by flow cytometric analysis. Exposure of A2780 and HepG2 cells to THA for 48 hr resulted in a significant dose-dependent increase in apoptotic cells (Fig. 4A). At 40 µg/ml concentration, there was ∼10.6-fold (p<0.001) increase in the population of A2780 cells undergoing apoptosis compared to the untreated control cells (DMSO). Similar apoptosis index patterns were obtained when the HepG2 were exposed to THA (Fig. 4A, 4B). Ovary epithelial cell IOSE144 and normal liver cell QSG7701 were resistant to THA, under 30 µg/ml exposure, the total apoptotic cells were significantly lower when compared to the A2780 and HepG2 cells (Fig. 4C). The THA induced cell apoptosis can partially be blocked by pan-caspase inhibitor z-VAD-fmk (Fig. 4D). In order to confirm that the cancer cell death was caused by apoptosis, we also performed a DNA fragmentation assay and found that THA dose dependently increased apoptotic DNA fragmentation in A2780 and HepG2 cells 48 hr after THA treatment (Fig. 4E). These data showed that THA is also a potent inducer for apoptosis and it exerts its growth inhibition by inducing apoptosis.

### THA induces a G2/M cell cycle arrest

**Figure 5:**
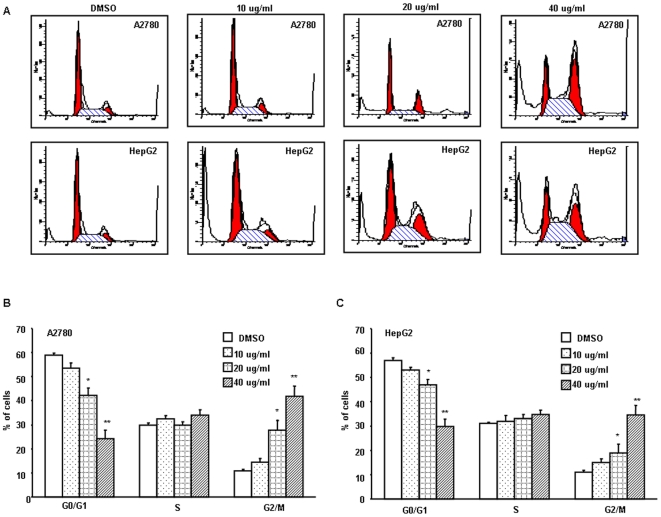
THA induces G2/M cell cycle arrest in A2780 and HepG2 cells. A2780 and HepG2 cells were treated with 0∼40 µg/ml THA for 48 hr and the cell cycle distribution was analyzed by the Becton Dickinson FACSCaliburr. (A) Histograms of the cell cycle distribution. (B, C) The percentage of cells in different phases. The results are the mean data from triplicate repeats under the same conditions (*, P<0.05; **, P<0.01 versus the control).

THA is structural similar to ursolic acid, a triterpenoid that also exhibits a potent anti-proliferative effect in human cancer cells. Previous, it has been shown that ursolic acid treatment caused a G1 arrest [30]. Thus, we also examined the effect of THA treatment on cell cycle progression. We found that THA induced a dose-dependent cell cycle arrest in the G2/M phase of A2780 and HepG2 cells (Fig. 5A). Treatment of A2780 cells with THA at the concentration of 40 µg/ml for 48 hr resulted in significant cell cycle arrest at G2/M phase which was accompanied by a decrease in G0/G1 phase cells. Also, a slight increase in the S phase population was observed after each THA treatment. The amount of G2/M-phase cells was tripled in the treated A2780 cells, compared to the controls, whereas G0/G1-phase cells were reduced by nearly 56% (p<0.01; Fig. 5B). A similar result was also observed in HepG2 cells (Fig. 5C). Thus, THA and ursolic acid, despite their structural similarity, have different effects on cell cycle progression, suggesting that they exert their growth inhibitions via different mechanisms.

### THA alters the expression of apoptosis-related proteins

**Figure 6:**
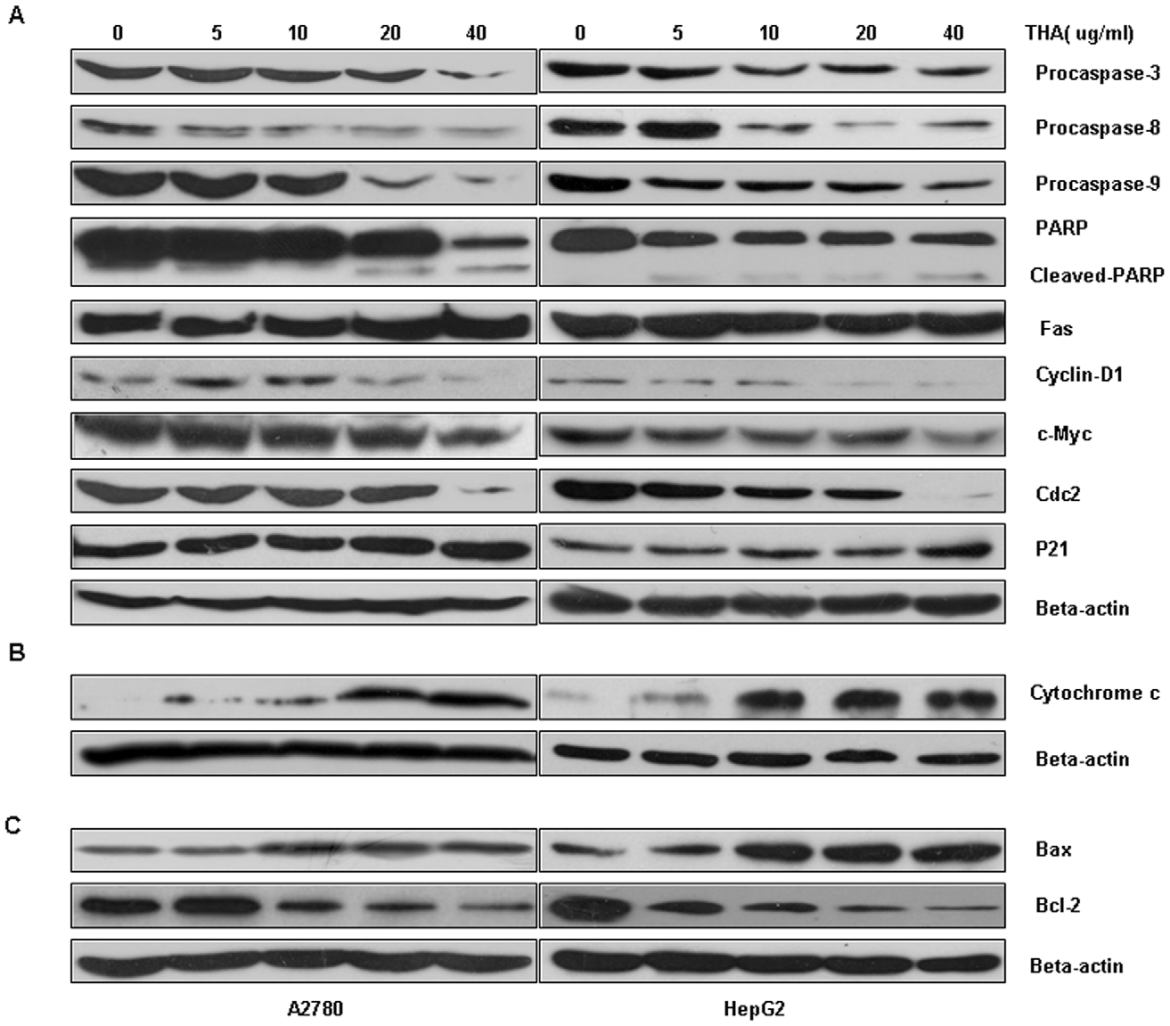
THA changes the expression of proteins related to apoptosis and cell cycle arrest. A2780 and HepG2 cells were exposed to various concentrations (0, 5, 10, 20 and 40 µg/ml) of THA for 48 hr, and protein levels were determined by Western blot. (A) The expression changes of caspases, PARP, c-Myc and cell cycle related proteins. (B) The release of cytoplasmic Cytochrome c from mitochondrion induced by THA treatment. (C) The expression of the apoptosis related proteins Bax and Bcl-2.

To examine the possible pathway of anti-proliferative, pro-apoptotic, and cell cycle arrest upon treatment with THA, we compared the expression of various proteins involved in these processes by Western blot. Consistent with its effect on apoptosis, THA induced changes in the expression of apoptosis related proteins in A2780 and HepG2 cells. The expression of procaspase-3, -8, -9, and c-Myc were clearly decreased in a dose-dependent manner. Cyclin D1 expression was slightly decreased, but there was no change for the Fas expression (Fig. 6A). Moreover, the PARP cleavage was induced, where the 116 kd protein was cleaved to an 89 kd catalytic fragment (Fig. 6A). THA also induced changes in the expression of cell cycle regulatory proteins, and the expression level of Cdc2 were decreased clearly (Fig. 6A). In addition, cytosolic release of Cytochrome c from mitochondrion was detected in both cells (Fig. 6B). Moreover, A2780 and HepG2 cells exposed to THA showed a dose-dependent reduction in the level of Bcl-2 protein, with a concomitant increase in the level of Bax and the Bax/Bcl-2 ratio, compared with the control cells (Fig. 6C).

## Discussion

Triterpenoids represent an emerging class of promising anticancer agents [7], [9]. Here we have reported the isolation of 2α, 3α, 19β, 23β-tetrahydroxyurs-12-en-28-oic acid (THA), a novel triterpenoid compound from the leaves of *Sinojackia sarcocarpa*. We showed that this compound exhibited a high degree of cancer-selectivity in its cytotoxic effect in both *in vitro* and *in vivo* settings. Together, our data have strongly demonstrated that THA represents an attractive candidate as an anticancer agent. It is a new addition to the class of triterpenoid compounds which possess anti-proliferative/anticancer activities.

To elucidate the anti-cancer mechanism of THA, we investigated cell apoptosis, the cell cycle and the expression of the proteins following THA treatments. Our data showed that THA treatment induced strong cell apoptosis in both A2780 and HepG2 cells (Fig. 4). Apoptosis is an active physiologic process of cellular self-destruction, with specific biochemical changes in the nucleus and cytoplasm via either the intrinsic (mitochondrial) or extrinsic pathways [31], [32]. The intrinsic pathway is characterized by the release of Cytochrome c from the mitochondria to the cytoplasm and activation of the caspase-dependent and -independent pathway [33]. In this study, we found that THA treatment induced the release of Cytochrome c from the mitochondria in a dose-dependt manner, resulting in the activation of procaspase-9 and procaspase-3. Moreover, these changes are associated with a significant increase in the cleavage and activation of PARP, a target protein of caspase-3 (Fig. 6A, 6B). Together, these data indicate the involvement of intrinsic pathway in THA-induced apoptosis. THA is structurally related to ursolic acid, a major member of the triterpenoid family with anti-proliferative/anticancer activities that have already been reported [34], [35]. Interestingly, both agents significantly suppress the expression of Bcl-2 and increase the expression of Bax. Bcl-2 and Bax are important members linked to mitochondrial control during apoptosis: Bax, a pro-apoptotic member, favors the leakage of apoptogenic factors from mitochondria, and Bcl-2, an anti-apoptotic member, negatively regulates this leakage [36], [37]. The changes in the levels of Bcl-2 and Bax expression are consistent with those of caspases and Cytochrome c, supporting the conclusion that the intrinsic pathway is the major mechanism of apoptosis following THA exposure. However, we have noted that despite their structural similarity, THA and ursolic acid appears to have different effects on cell cycle progression. For examples, THA causes a G2/M arrest, while ursolic acid induces primarily a G1 arrest [30]. This difference strongly suggests that these two drugs may exert their growth suppression on human cancer cells via distinctive mechanisms. Intriguingly, we have also noticed a slight increase in the S phase population following THA treatments, indicating that THA also has an effect on S phase progression. This has also raised the possibility that the G2/M arrest and growth suppressive effect of THA could have originated, at least partly, from its effect on S phase progression. Future experiments will shed new lights on the underlying mechanisms or potential targets of these various types of triterpenoids, and will hopefully promote the development of these agents as anticancer drugs.

## Supporting Information

Figure S1 **The isolation process of 2α, 3α, 19β, 23β-tetrahydroxyurs-12-en-28-oic acid (THA) from the leaves of** ***Sinojackia sarcocarpa*** **.**
(TIF)

Figure S2 **THA did not react with the MTT assay reagents.** Various concentrations of THA were added in the 96-well plate in the absence of cells, MTT assay was performed and the absorbance of 550 nm was measured.
(TIF)
