# Supplementary figures and images for: Anticancer Activity of 2α, 3α, 19β, 23β-Tetrahydroxyurs-12-en-28-oic Acid (THA), a Novel Triterpenoid Isolated from Sinojackia sarcocarpa
Source: PLoS One. 2011 Jun 10;6(6):e21130. doi: 10.1371/journal.pone.0021130 (PMC3112233; doi:10.1371/journal.pone.0021130)

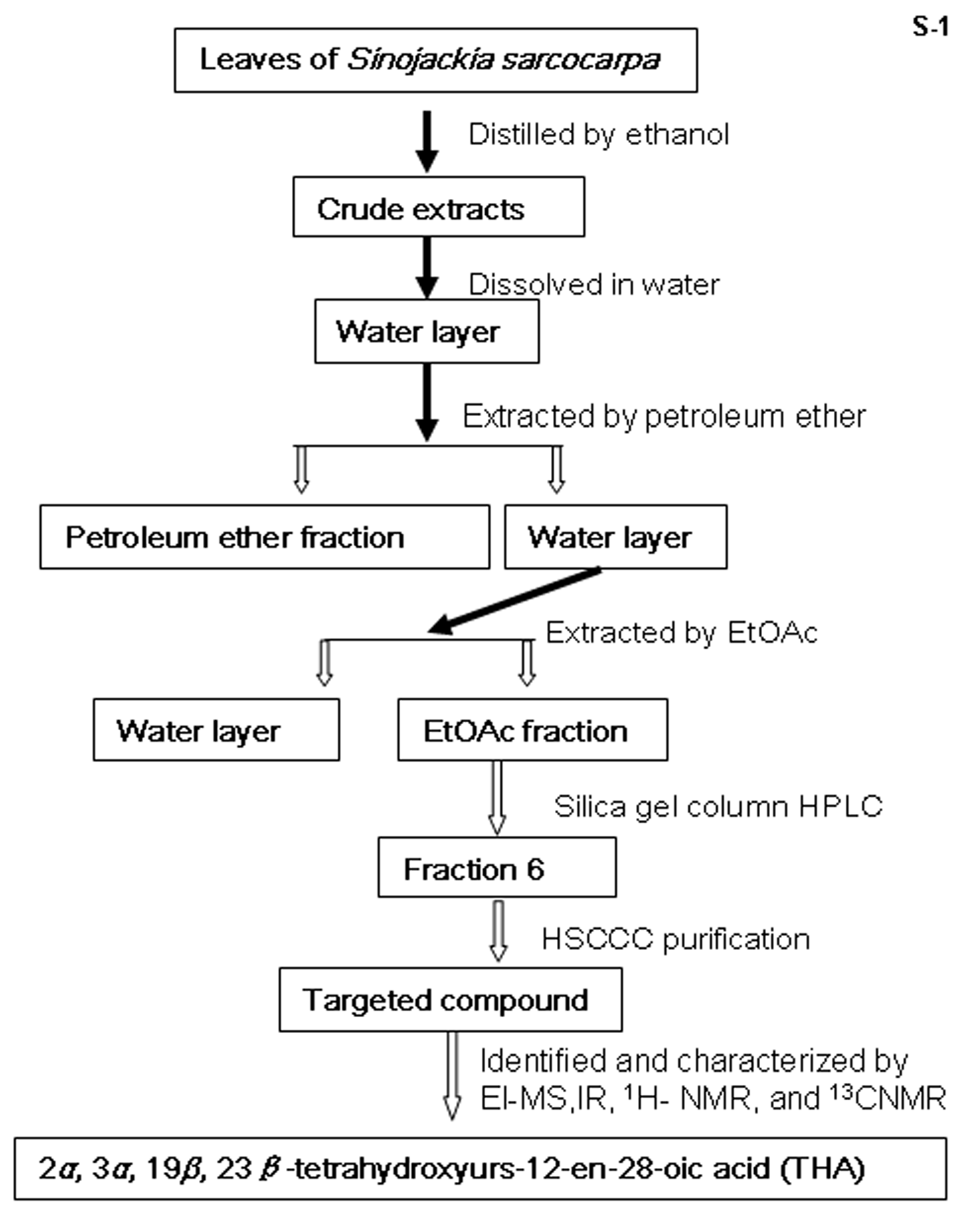

Supplement: Figure S1 — The isolation process of 2α, 3α, 19β, 23β-tetrahydroxyurs-12-en-28-oic acid (THA) from the leaves of Sinojackia sarcocarpa . (TIF) [file pone.0021130.s001.tif]

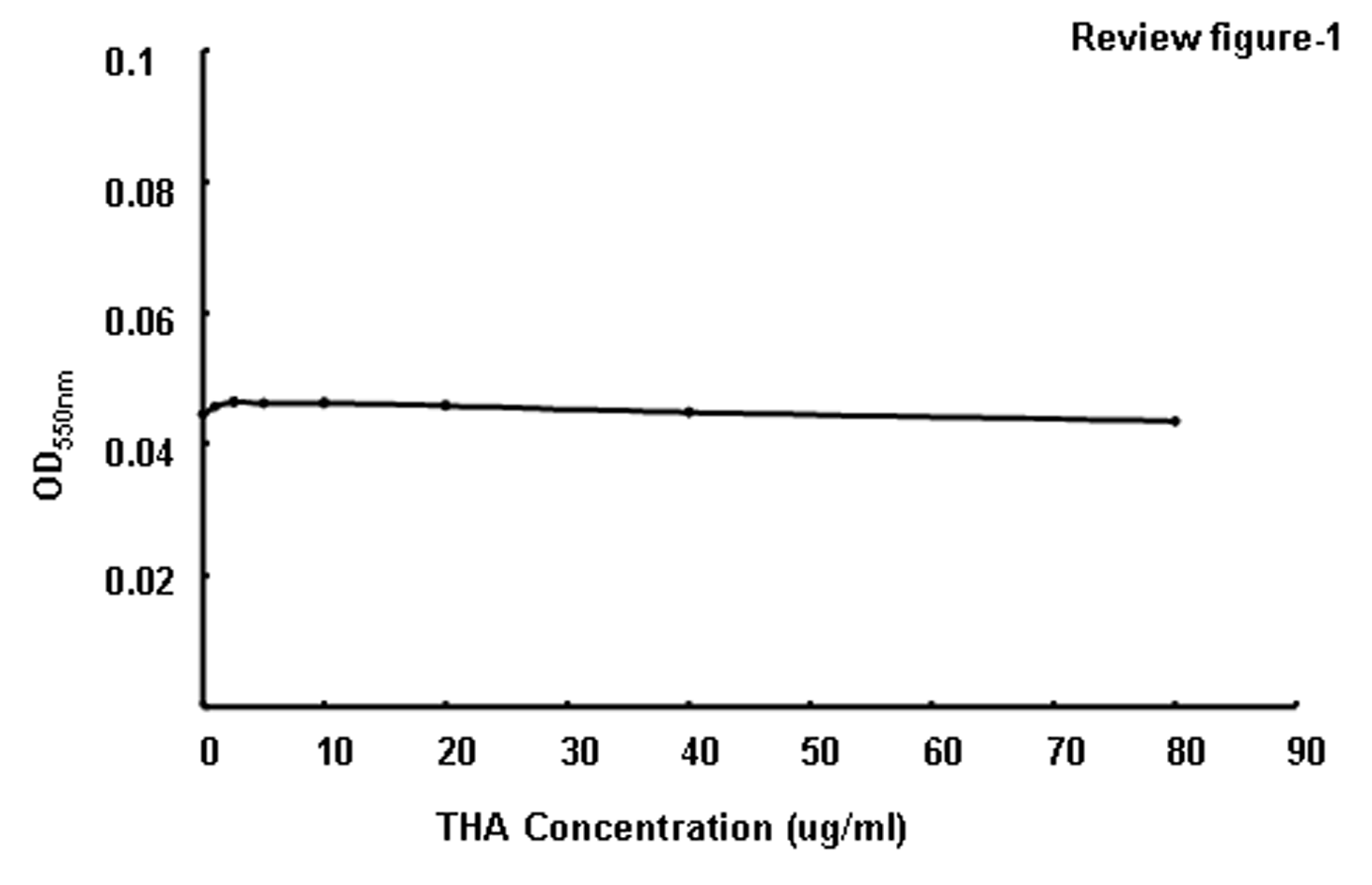

Supplement: Figure S2 — THA did not react with the MTT assay reagents. Various concentrations of THA were added in the 96-well plate in the absence of cells, MTT assay was performed and the absorbance of 550 nm was measured. (TIF) [file pone.0021130.s002.tif]
